# Prescribing for Substance Misuse: Alcohol Detoxification in Adult Mental Health Inpatient Services. The National Prescribing Observatory for Mental Health (POMH-UK) Quality Improvement Programme: 14c

**DOI:** 10.1192/bjo.2025.10550

**Published:** 2025-06-20

**Authors:** Fareeba Anwar, Haroon Siddiq, Roisin Nuttall

**Affiliations:** 1 Merseycare.nhs.uk, Warrington, United Kingdom; 2 Cheshire and Wirral NHS Trust, Chester, United Kingdom

## Abstract

**Aims:** To help specialist mental health Trusts/healthcare organisations improve their prescribing practice. To assess the Trusts’ alcohol detoxification practices, benchmark against the national average performance and to compare results to the previous audit of 2016. This was the second re-audit in the cycle.

**Methods:** The audit included any person (Male and Female) admitted to an acute adult or psychiatric intensive care ward, or a specialist inpatient drug or alcohol unit, who underwent alcohol detoxification (assisted alcohol withdrawal) whilst an inpatient. Patients identified via RiO, EPMA, Pharmacy Databases and Ward/Team caseloads. The final sample consisted of 80 patients, 20 patients from each of the 4 boroughs (Warrington, Halton, Kn owsley, Wigan).

Data was collected in May 2021 via clinical audit days over Microsoft Teams, checked for quality twice by the audit leads and inputted by the Medicines Management Team in June 2021.

Data was analysed by the national POMH team followed by the production of a national and trust level report. The findings of the audit were then presented at various forums.

**Results:** Five POMH-UK Standards were chosen for the audit that were divided in 12 criteria to assess results against. In addition, three “targets” for assessing alcohol intake at admission and follow up following detoxification process were also selected for the audit.

Four of twelve standard criteria (assessment of.iver function test and glucose tests, prescription for acute alcohol withdrawal and prescribing parenteral thiamine) and all the three targets (measurement of alcohol breath test at initial assessment, relapse prevention treatment and referral to specialist alcohol services for follow up) indicated less than 60% compliance with standards and a lower compliance than the national sample.

For all targets, practices across the trust were variable.

**Conclusion:** There is a need for developing guidance, detailing professional responsibilities and aligning procedures and documents across Mersey Care Foundation Trust to create a single standard method for alcohol detoxification. This includes developing a standard junior doctor admission checklist for both assessment and monitoring and raising the importance of assessment for alcohol detox at Junior doctor induction programme. It highlighted the need to consider alcohol breath test as part of the initial assessment at admission and including the required blood tests in routine admission blood investigations. The financial implications of this were also considered. The process emphasised the need for dissemination of results to all staff, focussing mainly on inpatient staff.

